# AF-DHNN: Fuzzy Clustering and Inference-Based Node Fault Diagnosis Method for Fire Detection

**DOI:** 10.3390/s150717366

**Published:** 2015-07-17

**Authors:** Shan Jin, Wen Cui, Zhigang Jin, Ying Wang

**Affiliations:** 1 School of Electronic Information Engineering, Tianjin University, Tianjin 300072, China; E-Mail: hellohiten@126.com; 2 Fire Brigade of Hexi District, Tianjin 300222, China; 3 School of Management, Tianjin Polytechnic University, Tianjin 300387, China; E-Mail: CuiWen0911@163.com; 4 Nankai Hospital of Traditional Chinese Medicine, Tianjin 300102, China; 5 Guangxi Experiment Center of Information Science, Guilin 541004, China

**Keywords:** wireless sensor networks, node fault diagnosis method, fire detection, adaptive, Discrete Hopfield Neural Network, fuzzy C-means algorithm, fuzzy inference

## Abstract

Wireless Sensor Networks (WSNs) have been utilized for node fault diagnosis in the fire detection field since the 1990s. However, the traditional methods have some problems, including complicated system structures, intensive computation needs, unsteady data detection and local minimum values. In this paper, a new diagnosis mechanism for WSN nodes is proposed, which is based on fuzzy theory and an Adaptive Fuzzy Discrete Hopfield Neural Network (AF-DHNN). First, the original status of each sensor over time is obtained with two features. One is the root mean square of the filtered signal (FRMS), the other is the normalized summation of the positive amplitudes of the difference spectrum between the measured signal and the healthy one (NSDS). Secondly, distributed fuzzy inference is introduced. The evident abnormal nodes’ status is pre-alarmed to save time. Thirdly, according to the dimensions of the diagnostic data, an adaptive diagnostic status system is established with a Fuzzy C-Means Algorithm (FCMA) and Sorting and Classification Algorithm to reducing the complexity of the fault determination. Fourthly, a Discrete Hopfield Neural Network (DHNN) with iterations is improved with the optimization of the sensors’ detected status information and standard diagnostic levels, with which the associative memory is achieved, and the search efficiency is improved. The experimental results show that the AF-DHNN method can diagnose abnormal WSN node faults promptly and effectively, which improves the WSN reliability.

## 1. Introduction

As user demands for network reliability and sustainability increase, research and innovation in hardware design, calculation processing, wireless communication, network protocols, and the energy efficiency of nodes are issues that continue to be raised in Wireless Sensor Network (WSN) studies. WSNs are widely utilized in the military and civilian fields. Because sensor nodes can integrate multiple functions including detection, distributed computation, and preliminary data fusion, the probability of node module failures is increasing. Consequently, rapid and correct fault diagnosis has become a hot topic in recent years. The main focus of this paper is on module failure detection, combining various faults of function modules, and analysis of the specific status of faulty nodes.

According to the characteristics of fault diagnosis, several problems must be resolved. They are: (1) the expression of multi-function module faults on the same node; (2) an actual model to establish real scenarios; and (3) accurate detection of the location of faulty nodes.

The existing published research offers few effective methods to solve the above problems, and the schemes are explained in the related work section. In this paper, first the characteristics of the samples are extracted and attention is paid to ANN knowledge acquisition with fuzzy inference. Then, a kind of Adaptive Fuzzy Discrete Hopfield Neural Network (AF-DHNN) method is designed. First, the original detected data are obtained with two features. Secondly, expression of module states with membership functions, data detection of each module, initial determination of the states of nodes and modules, and part of the early fault diagnosis process are actualized by fuzzy inference. Thirdly, the multi-dimensional central points are found by c-means clustering, sorting and classification, and an adaptive clustering rating system is established. Fourthly, according to the above rating system, the value set after fuzzy inference is two-value processed to obtain real node and module states with the characteristics of a single layer discrete Hopfield neural network. Finally, the performance of AF-DHNN is verified in a high-rise building fire model.

The main contributions of this paper include: (1) the expression of failure phenomena based on different module functions; (2) the role of a fuzzy clustering method through adaptive fault diagnosis for the establishment of standards; (3) establishment of a twice-alarmed mechanism for fault diagnosis based on the characteristics of the distributed computation in WSNs, which mechanism can consolidate the fault information and improve the detection efficiency; (4) an adaptive diagnostic standard is created, which can be an accurate judge of node state, and a solution to the original problem of excessive fault detection.

This article is arranged as follows: in Section 2, related work is introduced. In Section 3, the algorithm to determine the diagnosis method is provided. In Section 4, the mathematical model and simulation environment are established, and the implementation of the solutions is presented to verify the properties and numerical analysis through an actual fire environment simulation. Finally, the conclusions of the paper are presented in Section 5.

## 2. Related Works

Existing works on WSN node fault diagnosis have mainly concentrated on theoretical research, routing information or node data fault diagnosis [1,2,3,4], mainly based on Bayesian decision, distributed fault detection, neural network algorithms and so on.

### 2.1. The Diagnostic Methods Based on Traditional Probability Theory

In [5], a data change rate method was proposed. The normal data change rate was contained in a pick data zone, which for anomalies found and outliers rejection changed with the change rate and adjusted the width of pick data zone based on the sampling data. In [6], a fault diagnosis method for WSN sensors based on Bayes decision theory was proposed. The basic principle of this algorithm was used for fault location and repair decisions in the modules of each node. Moreover, in carrying out the analysis using Bayes decision theory, the collection of historical fault information from WSNs is essential. Then, a prior probability of fault status is detected, and a decision based on the real-time sign state made for the posterior probability. Finally, according to the Bayesian decision criteria, whether or not a node failure had occurred was determined. In addition, this method based on Bayesian decision theory has little communication costs and energy consumption. If the node fault sample decision table with historical fault information were more reliable, the accurate fault diagnosis rate would be higher. Hence, it is a method applicable for fault diagnosis in WSNs which have limited node energy. On the other hand, the method is excessively dependent on the availability of WSN node fault sample decision tables, historical fault information, and prior probabilities of various faults that can be used as statistics for nodes are essential.

### 2.2. Distributed Fault Detection Methods

Because of the diversified functions of WSN nodes and the increasing number of nodes used in modern networks, the demand for distributed fault diagnosis has increased. In [7], distributed fault detection (DFD) was put forward as a fault diagnosis method for WSN sensors. This method tests the relationships between neighboring nodes for fault diagnosis. Similarly, another method which compared the results of nodes with the adjacent ones was proposed in [8]. Faulty nodes were identified by the diffusion of an established decision strategy. In [9], because of the differences between multi-sensors located in an identical space and their relevant characteristics, a fault detection algorithm based on the DFD algorithm was proposed. Consequently, the fault detection accuracy was increased and this method was adaptable to WSNs with discrete node distribution and high fault rates. In [10], a fault detection algorithm based on interspace interdependency and time redundancy was proposed. The algorithm had high fault tolerance ability and low false alarm rates, but it consumed more energy to spread the initial state information to each node. In [11], an improved DFD algorithm, which suggested the DFD algorithm, is so rough that it could not determine the final network state proposed. Then, the conditions were modified to improve the ability of the DFD algorithm, but the high energy consumption remained as an urgent problem to be solved. In [12], a fault diagnosis algorithm based on clustering was proposed, which utilized the cluster head node to detect faulty nodes. It utilized the optimal threshold to improve the detection accuracy and it also reduced the impact of the sensor fault probability. However, with this algorithm the energy consumption was unbalanced.

### 2.3. The Diagnosis Method Based on Artificial Intelligence

Recently, the artificial intelligence method was introduced for fault diagnosis applications. Because neural networks have convenient learning and data structure optimization training, the WSN node status could be utilized in a larger range, with more efficiency, and in a multi-function environment. In [13], a fault diagnosis method for WSN sensors based on a neural network was proposed. It solved several problems, including those of redundant information and limited node energy. Moreover, it is based on a neural network for WSN node fault diagnosis with significant uncertainty, which could calculate results rapidly and accurately, and achieve more robustness and better applicability, but a new problem arising from the large amount of calculations needed is the poor real-time performance of the algorithm. In [14], an engine fault diagnosis algorithm based on an intelligent methodology was proposed. It utilized the multiple model approach and auto associative neural networks (AANNs) to create a fault detection and isolation (FDI) scheme. Besides, it provided a new integrated solution to concurrently provide component fault detection and isolation. In [15], the safe operation of hydraulic generator units (HGUs) was the most important feature of the proposed fault diagnosis. In the algorithm, the macroscopic Euler number (ME), fuzzy convex-concave feature (FCC) and boundary-layer feature (BL) were proposed for three different aspects: boundary, structure and region. The most effective and comprehensive image information was fully integrated by a feature vector composed of ME, FCC and BL. Moreover, according to the feature vector, the probability neural network (PNN) was utilized as the classifier. Finally, a swarm intelligence algorithm method based on the characteristic simple rules and fast convergence speed of the particle swarm optimization algorithm, to carry out optimization of node data, get a threshold range, and judge if the data satisfied the threshold range to determine a node status using a Gaussian distribution, was introduced in [16].

### 2.4. Other Diagnostic Methods

Over the same period, some other diagnostic methods have been developed. An identification algorithm for faulty nodes was proposed in [17]. In [18], an energy efficient fault-tolerant technology for fault detection in wireless sensor networks was proposed. In [19], a wireless sensor network node fault diagnosis algorithm based on rough set theory was proposed. In [20], a sensor crushing failure recognition algorithm was proposed. These methods all showed improved fault finding and decision efficiency, but most of them did not utilize the redundant information in the time and space range and they suffered from low correct diagnosis rates, poor timeliness and other defects.

The Hopfield neural network is a single-layer feedback network which has more computing power than the BP network [21,22], and its most prominent advantage is its strong associative memory and optimization capabilities. It is typically utilized in resolving complex optimization problems such as voting analysis [23] and TSP [24] and when determining environmental parameters, it is easy to get the optimal solution quickly. In this paper, we combine fuzzy clustering, neural networks, and fuzzy inference and the detected data is collected. In addition, the node state is judged and fault feedback is taken into the research category. Finally, the above problems are resolved by establishing a high precision, strong reliability and practical method.

## 3. The Method Based on AF-DHNN

**Figure 1:**
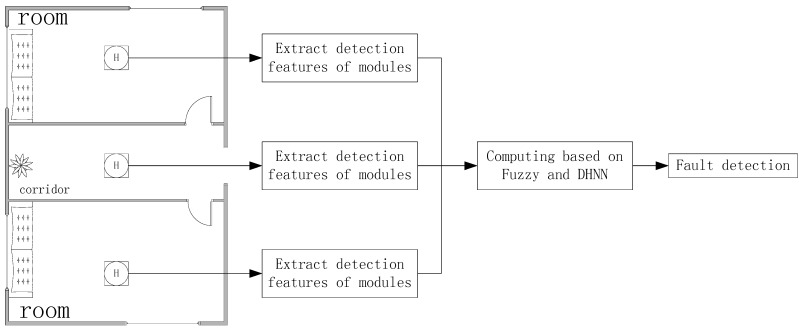
Flowchart of the proposed method.

Figure 1 displays the flowchart of the proposed method based on the AF-DHNN algorithm. Firstly, two features are extracted from the signals measured by sensors at different locations in a building. These features are the root mean square of the filtered signal (FRMS) and the normalized summation of the positive amplitudes of the difference spectrum between the measured signal and the healthy node one (NSDS). Secondly, a fuzzy inference operator is established to detect the single-node status at each node. Reasonable membership functions and fuzzy rules are set up to map the actual state of a node and failure problems. At the same time, the pre-alarmed set of sensors with the heaviest damaged nodes is actualized. Thirdly, the fuzzy c-means method is adopted to find the center of various failure problems, and an adaptive clustering rating system is established. Finally, the original values are taken into DHNN with the diagnosis standard and iterations [25,26] after applying FCMA and the Sorting and Classification algorithm [27,28]. Then, nodes are classified according to their different fault conditions by clear fault reasons, number and location for convenient statistics and maintenance.

### 3.1. Selection of Two Features for Detected Signals

First of all, the original data samples are obtained. As mentioned above, two features are extracted from the detected signals, which include the extent of flue gas dimming, temperature, and communication status. Besides, FRMS and NSDS adjust the inputs of the AF-DHNN, which are specially designed for fault detection of fires in buildings. *T* is the detected duration each time.

#### 3.1.1*.* FRMS

This feature is generated by calculating the root mean square of the filtered signal instead of the original signal and it is defined as:

(1) $${\text{FRMS}}_{x_{org},\;y_{org}}={\left\{x\left(t\right),\;y\left(t\right)\right\}}_{T}=\forall \sqrt{\frac{1}{T}\overset{T}{\underset{t=1}{\sum }}{\left(f_{x_{org},\;y_{org}}\left(t\right)\right)}^{2}}$$

where $f_{x_{org},\;y_{org}}\left(t\right)\left(t=1,\;\ldots ,\;T\right)$ is the *t*th time point data of the filtered signal. The filtered signal is produced by removing the regular meshing elements from the original detected signals, in which the terms $x_{org}$ and the $y_{org}$ separately stand for the flue gas dimming extent and temperature. Then, x(t), y(t) are the normal probabilities of flue gas dimming extent and temperature detected after time *T*, respectively.

#### 3.1.2*.* NSDS

This feature is developed by normalizing the summation of the positive amplitudes of the difference spectrum between the communication signal measured on a module whose health condition is unknown and the signal measured on a healthy module. It is expressed as:

(2) $${\text{NSDS}}_{z_{org}}={\left\{z\left(t\right)\right\}}_{T}=\forall \frac{\sum_{t=1}^{T}z_{m}\left(t\right)\;}{T}$$

(3) $$\{\begin{array}{c} z_{m}\left(t\right)=1-z_{s}\left(t\right),\;\;if\;z_{s}\left(t\right)<1\\ z_{m}\left(t\right)=0,\;\;\;\;\;\;\;\;\;if\;z_{s}\left(t\right)=1 \end{array}$$

where $z_{m}\left(t\right)\left(t=1,\;\ldots ,\;T\right)$ denotes the *t*th normal probability, and the $z_{s}$is the abnormal one. Then, z(t) is the normal probability of communication status detected after time *T*.

### 3.2. Fuzzy Inference Operator

After samples are collected, the original data are subjected to fuzzy inference processing with a fuzzy controller [29,30]. Fuzzy set theory, expert systems, and control theory are used for obtaining the fuzzy solutions of problems with complex processes and indirect modeling and also to initially diagnose each WSN node’s status.

**Figure 2:**
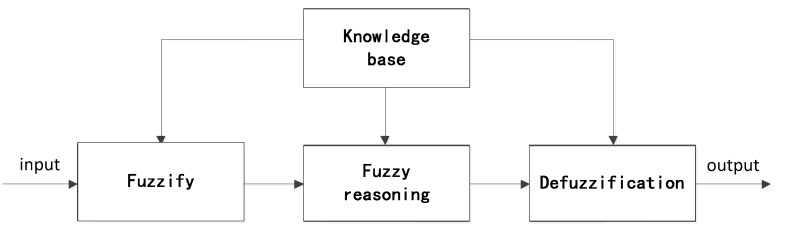
The basic principle of the fuzzy controller.

As shown in Figure 2, the fuzzy controller involves fuzzification, knowledge base, fuzzy inference and defuzzification steps.

#### 3.2.1*.* Basic Design

The structure of nodes based on the WSN distributed computation should be concise to establish a standard (Mamdani) model for analyzing the state of a single node. Unified sensor nodes are used in this paper. Each one is composed of a smoke detector, a temperature detector, a main communication module, and a standby communication module. Multi-input and single-output are utilized to simplify the subsequent analysis. As shown in Equations (4) and (5), the variables are normal probability sets including flue gas dimming extent *x*, ambient temperature *y*, communication module *z*, and output node status *s* in time *T*. The input discourses are *U*, *V*, and *W*. The output discourse is $Q=\left\{D_{1},D_{2},D_{3},D_{4}\right\}$:

(4) $$\{\begin{array}{c} x=\int_{t=1}^{T}x\left(t\right)dt\\ y=\int_{t=1}^{T}y\left(t\right)dt\\ z=\int_{t=1}^{T}z\left(t\right)dt \end{array}$$

(5) $$\{\begin{array}{c} A=\overset{\;}{\underset{U}{\int }}\frac{{\text{μ}}_{A}\left(x\right)}{x}\\ B=\overset{\;}{\underset{V}{\int }}\frac{{\text{μ}}_{B}\left(y\right)}{y}\\ C=\overset{\;}{\underset{W}{\int }}\frac{{\text{μ}}_{C}\left(z\right)}{z} \end{array}$$

The single node states used in the scheme are listed in Table 1.

**Table 1:** Single node state fuzzy set.

Output State	Flue Gas Dimming Extent	Ambient Temperature	Communication Module	Node State
normal	*A_1_*	*B_1_*	*C_1_*	*D_1_*
abnormal	*A_2_*	*B_2_*	*C_2_*	*D_2_*, *D_3_*, *D_4_*

In which,

*A_1_*——Smoke sensing module is normal;
*A_2_*——Smoke sensing module is abnormal;
*B_1_*——Temperature sensing module is normal;
*B_2_*——Temperature sensing module is abnormal;
*C_1_*——Enable the main communication module;
*C_2_*——Enable the standby communication module;
*D_1_*——Overall state of node is normal;
*D_2_*——Smoke sensing module fault;
*D_3_*——Temperature sensing module fault;
*D_4_*——Node main communication module fault, switching to standby communication module.

#### 3.2.2. Membership Function and Rules

**Figure 3:**
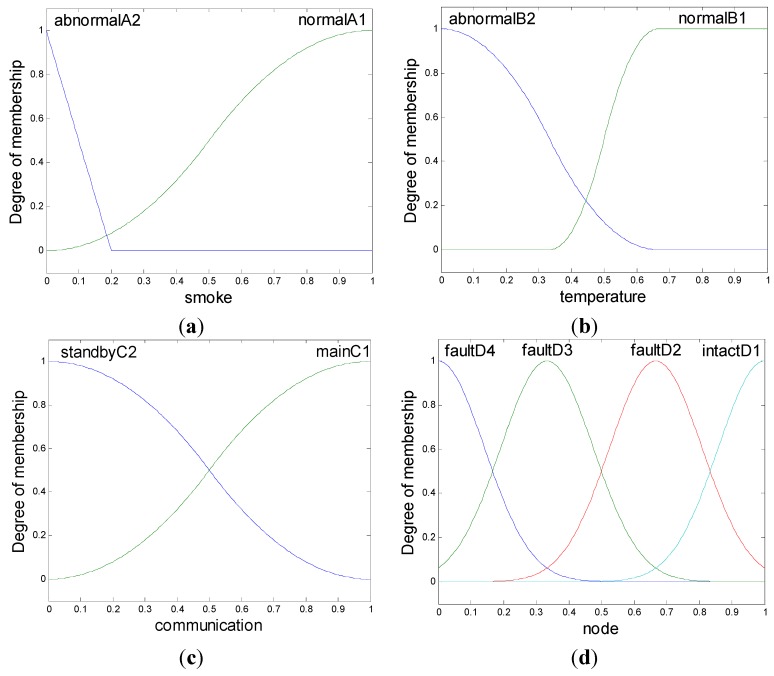
Membership functions. (**a**) smoke; (**b**) temperature; (**c**) communication; (**d**) node.

After the basic fuzzy inference design, the regulations of single items and the rules need to be established. The membership functions used in the scheme are shown in Figure 3. Because smoke spreads easier, faster, and is more dangerous than heating, the membership detected smoke function is more sensitive, and the abnormal status is detected more simply. Also, its abnormal range is more narrow and can be kept separate from cigarette and cooking fire effects. In contrast, the membership function of communication, which detects modules, is divided between identical function and structure.

**Figure 4:**
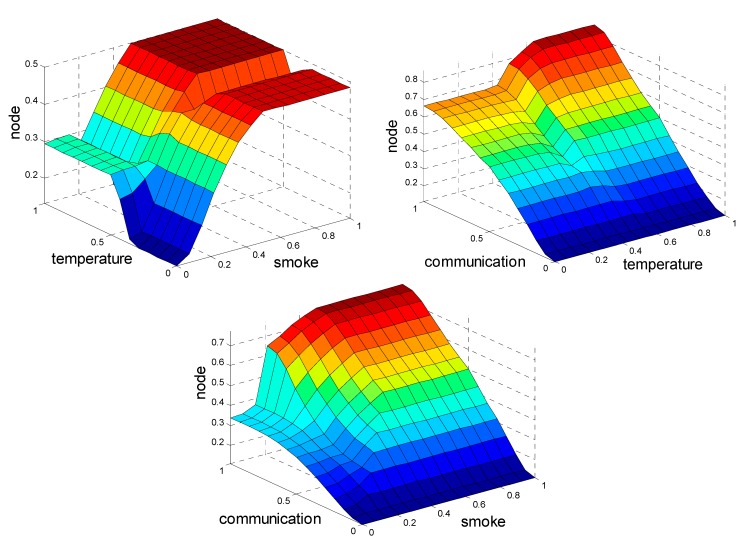
Fuzzy surface.

In addition, the membership function of the node status is divided according to criticality. The distribution of items from safe to dangerous are intact, temperature detector module faults, smoke faults, and main communication faults. Hence, as shown in Figure 4, the fuzzy rules are settled with the distribution items. The fuzzy rules are:

*R_1_*: *IF x is A_1_ and y is B_1_ and z is C_1_ THEN s is D_1_*
*R_2_*: *IF x is A_1_ and y is B_1_ and z is C_1_ THEN s is D_2_*
*R_3_*: *IF x is A_2_ and y is B_1_ and z is C_1_ THEN s is D_3_*
*R_4_*: *IF z is C_2_ THEN s is D_4_*

All nodes in this layer are fixed. They are labeled with the rules, indicating that they perform as simple multipliers. Then, the outputs of this layer represent the fuzzy strengths *ω_i_* of each rule and can be expressed as:

(6) $$o_{i}^{3}={\text{ω}}_{i}=u_{A_{i}}\left(x\right)u_{B_{i}}\left(y\right)u_{C_{i}}\left(z\right),\;i=1,\;2,\;3,\;4$$

specific to each rule, which is also expressed as:

(7) $$\{\begin{array}{c} {\text{ω}}_{1}=u_{A_{1}}(x)u_{B_{1}}(y)u_{C_{1}}(z)\\ {\text{ω}}_{2}=u_{A_{1}}(x)u_{B_{2}}(y)u_{C_{1}}(z)\\ {\text{ω}}_{3}=u_{A_{2}}(x)u_{B_{1}}(y)u_{C_{1}}(z)\\ {\text{ω}}_{4}=u_{C_{2}}(z) \end{array}$$

#### 3.2.3. Normalization

All nodes in this layer are also fixed. They are labeled as *N*, indicating that they play a normalization role on the fuzzy strengths from the previous layer. The normalization factor is calculated as the sum of all weight functions. Then, the outputs of this layer, the so-called normalized fuzzy strengths, can be represented as:

(8) $$o_{i}^{4}={\bar{\text{ω}}}_{i}={\text{ω}}_{i}/\overset{4}{\underset{i=1}{\sum }}{\text{ω}}_{i},\;i=1,\;2,\;3,\;4.$$

#### 3.2.4. Output Membership Function

After fuzzy adjustment, the original data has the ability to introduce the preset status of each node and module. In this layer, the status is transformed to be discrete and explicit. At first, the weighted average (centroid) method is utilized. As shown in Equation (9), the clear data symbol is *S*, in which $S=s_{fuzzy}\left(x,\;y,\;z\right)\;$ and ${\text{μ}}_{D_{i}}$ stands for the above fuzzy rules *R_i_*:

(9) $$O_{1}=S\;=\left\{f^{5}\left(s\right)\right\}=\left\{\frac{\sum_{i=1}^{n}s_{i}{\text{μ}}_{D_{i}}\left(s_{i}\right)}{\sum_{i=1}^{n}{\text{μ}}_{D_{i}}\left(s_{i}\right)}\right\}$$

Then, the average status of all nodes is taken as a rough standard for selecting the heaviest damaged ones the first time, in which *alarm*^1^ is utilized to complete it:

(10) $$o_{i}^{5}=\text{β}=\frac{\sum_{i=1}^{n}f^{5}\left(s_{i}\right)}{n}$$

(11) $$if\;f^{5}\left(s\right)<n^{-\text{α}}\text{β}\;then\;alarm^{1}=\left\{f^{5}\left(s\right)\right\}$$

(12) $$\text{α}=log_{10}\left(n\right)$$

The result is in *O*_1_, which is used in the next step.

### 3.3. FCMA Operator

Now the sensors’ status is known, but it is too complex to select the faults. A clustering algorithm can solve this problem. Moreover, fuzzy clustering belongs to the unsupervised machine learning area, which classifies the input samples according to similar characteristics. In this paper, for resolving the problem with a discrete method and to easily fall into a local optimum in the traditional division algorithm, the global optimal solution model in a continuous space is established, which is named Fuzzy C-Means Algorithm (FCMA) [31,32].

In this algorithm, the sample set $O_{1}=S_{\;}=\left\{s_{1},{\text{ s}}_{2},\;\ldots {\text{s}}_{n}\right\},\;n$ is set as the number of classifying modules. The number of clustering centers is $c_{cluster}\in \left(1,\;n\right)$. In Equations (13) and (14), the objective function is redefined:

(13) $$J\left(S,\;E\right)=d_{P}^{2}\left(s_{i},\;e_{i}\right)=||s_{i}-e_{i}|{|}_{P}^{2}={\left(s_{i}-e_{i}\right)}^{T}P\left(s_{i}-e_{i}\right)$$

where $e_{\gamma_{i}}$ is a fuzzy partition matrix of *s*:

(14) $$e=\left[e_{1},\;e_{2},\;e_{3},\;\ldots e_{c_{cl}}\right]$$

is a vector of clustering centers, which have to be determined:

(15) $$d_{\gamma iP}^{2}=||s_{i}-e_{i}{\left|\right|}_{A}^{2}={\left(s_{i}-e_{i}\right)}^{T}P\left(s_{i}-e_{i}\right)$$

is a squared inner-product distance norm and:

(16) θ∈[1, ∞]

is a parameter that determines the fuzziness of the resulting clusters. The conditions for a fuzzy partition matrix are given as:

(17) $$\;e_{\gamma_{i}}\in \left[0,\;1\right],\;1\leq \text{γ}\leq c_{cl},\;1\leq i\leq n$$

(18) $$\overset{c_{cl}}{\underset{i=1}{\sum }}e_{\gamma_{i}}=1,\;1\leq i\leq n$$

(19) $$0<\overset{n}{\underset{i=1}{\sum }}e_{\gamma_{i}}<N,\;1\leq i\leq \text{γ}$$

The value of the objective Equation (13) can be seen as a measure of the total variance of *s_i_* from *e_i_*. The minimization of the objective Equation (13) as a non-linear optimization problem that can be solved by iterative minimization, simulated annealing or stationary points of Equation (13) is known as the fuzzy c-means algorithm (FCMA).

The stationary points of the objective Equation (13) can be found by adjoining the constraint Equation (18) to J(S, E) by means of Lagrange multipliers:

(20) $$J=\overset{c_{cl}\;}{\underset{\text{γ}=1}{\sum }}\overset{m}{\underset{i=1}{\sum }}{\left(e_{\gamma_{i}}\right)}^{\text{θ}}d_{\gamma_{i}P}^{2}+\overset{n}{\underset{i=1}{\sum }}\lambda_{i}\left[\overset{\text{γ}}{\underset{i=1}{\sum }}e_{\gamma_{i}}-1\right]$$

and by setting the gradient of *J* with respect to the fuzzy partition matrix *s*, the vector of clustering matrix *e* and λ to zero. Now, *if* $d_{\gamma_{i}P}^{2}>0,\;{\text{λ}}_{i},\text{ γ}$ and θ>1, then (s, e) may minimize the objective Equation (13) only if:

(21) $$\;\;\;\;\;\;e_{\gamma_{i}}=\frac{1}{\sum_{j=1}^{c_{cl}}{\left(d_{\gamma_{i}P}^{\;}/d_{\gamma_{j}P}^{\;}\right)}^{2}/\left(\text{θ}-1\right)},\;1\leq j\leq c_{cl},\;1\leq i\leq n$$

and:

(22) $$e_{i}=\frac{\sum_{i=1}^{n}{\left(e_{\gamma_{i}}\right)}^{\text{θ}}s_{i}}{\sum_{i=1}^{n}{\left(e_{\gamma_{i}}\right)}^{\text{θ}}};\;1\leq i\leq \text{γ}$$

This solution also satisfies Equations (17) and (19). Equations (21) and (22) are the first-order necessary conditions for stationary points of the objective Equation (13). The FCMA iterates through Equations (21) and (22). The sufficiency of the necessary Equations (21) and (22), as well as the convergence of the FCMA is proven in [33]. Before using the FCMA, the parameters are: the number of clusters, $c_{cl}$, the fuzziness exponent, θ, the termination tolerance, ϵ, and the norm-inducing matrix, *P*. The fuzzy partition matrix, *s*, must also be initialized. Note that the FCMA converges to a local minimum of the objective Equation (13). Hence, different initializations may lead to different results. FCMA is used to train and cluster the nodes states after fuzzy inference analysis of continuous time *t*, and compute the detection dimensions of the clustering center points $E=\{e_{\gamma_{i}}\}=\{D_{1}(x_{o},\;y_{o},\;z_{o}),\;D_{2}(x_{A},\;y_{A},\;z_{A}),\;D_{3}(x_{B},\;y_{B},\;z_{B}),\;D_{4}(x_{C},\;y_{C},\;z_{C}),\;\ldots ,D_{c_{cluster}}\}$.

Like in Equations (23)–(25), the class number based on the clustering center number of elements is determined adaptively. The grades standard is established from high to low. Besides, the clustering center set *D*, grading standard *L*, and the number of grades $c_{cl}=c_{cluster}+1$, which is also an element of *D*, are set up. The *descend* stands for descending order:

FCMA is used to train and cluster the nodes states after fuzzy inference analysis of continuous time *t*, and compute the detection dimensions of the clustering center points $E=\{e_{\gamma_{i}}\}=\{D_{1}(x_{o},\;y_{o},\;z_{o}),\;D_{2}(x_{A},\;y_{A},\;z_{A}),\;D_{3}(x_{B},\;y_{B},\;z_{B}),\;D_{4}(x_{C},\;y_{C},\;z_{C}),\;\ldots ,D_{c_{cluster}}\}$. As Equations (23)–(25), the class number based on the clustering center number of elements is determined adaptively. Then, the grades standard is established from high to low. Besides clustering center set *D*, grading standard *L*, and the number of grades $c_{cl}=c_{cluster}+1$, which is also element of *D*, are set up. The *descend* stands for descending order.

(23) $$D=\{0,D_{1},D_{2},D_{3},D_{4},\;\ldots ,D_{c_{cluster}}│S\}=[\begin{array}{c} x_{o},y_{o},z_{o}\\ x_{A},y_{A},z_{A}\\ x_{B},y_{B},z_{B}\\ x_{C},y_{C},z_{C}\\ \ldots \\ 0 \end{array}]$$

(24) $$D_{l}=sort\left(D,\;1,\;\prime descend\prime \right)$$

(25) $$L_{n}=\left[D_{l}\left(c_{cl},\;1\right),\;D_{l}\left(c_{cl},\;2\right),\;D_{l}\left(c_{cl},\;3\right)\right]=\left[L_{A_{c_{cl}}},\;L_{B_{c_{cl}}},\;L_{C_{c_{cl}}}\right]$$

**Algorithm 1.** Sorting and classification algorithm.
**Inputs: *S_i, j_, i* = 1, 2, … *n, j* = 1, 2, … *c_cl_ E =* {*e_γ_i__*}**
**Outputs: *Refresh S_i, j_, O*_1_**
**Initialize: *i, j***1.*S_i, j_ = *$S_{ij}^{T}$, *i* = 1, 2, … *n, j* = 1, 2, … *c_cl_*2.Establish grades standard:3.$D=\{0,D_{1},D_{2},D_{3},\ldots ,D_{C_{cluster}}│S\}$4.$D_{l}=sort\left(D,\;1,\;\prime descend\prime \right)$5.$L_{n}=\left[D_{l}\left(c_{cl},\;1\right),\;D_{l}\left(c_{cl},\;2\right),\;D_{l}\left(c_{cl},\;3\right)\right]=\left[L_{A_{c_{cl}}},\;L_{B_{c_{cl}}},\;L_{C_{c_{cl}}}\right]$6.Classified the data:7.For 1 ≤ *j* ≤ *c_cl_*; For 1 ≤ *i* ≤ *n*8.$\left\{ \begin{array}{c} \text{If}\;S_{i}(x,i,i)\geq L_{A_{i}}\;then\;D_{l}(i,1)=S_{i}(x,i,i)\;and\;S_{i}(x,i,i)=0\;else\;D_{l}(i,1)=0\\ \text{If}\;S_{i}(i,y,i)\geq L_{B_{i}}\;then\;D_{l}(i,2)=S_{i}(i,y,i)\;and\;S_{i}(i,y,i)=0\;else\;D_{l}(i,2)=0\\ \text{If}\;S_{i}(i,i,z)\geq L_{C_{i}}\;then\;D_{l}(i,3)=S_{i}(i,i,z)\;and\;S_{i}(i,i,z)=0\;else\;D_{l}(i,3)=0 \end{array}\right.$9.$Endif\;S_{i,j}=(D_{l}(i,1),D_{l}(i,2),D_{l}(i,3));\;O_{1}=\sum_{i=1}^{n}\sum_{j=1}^{C_{cl}}S_{i,j}$

Algorithm 1 is utilized for sorting the discrete detected data and distributing them into each group with grades *L*_1_ *to L_n_*. It is essential in the whole model, because the first action in the twice-alarmed mechanism of the proposed method attributes to the data collection with the lowest two grades. Plenty of the statuses without law become defined and regular to express the faults with the system.

### 3.4. Discrete Hopfield Network

**Figure 5:**
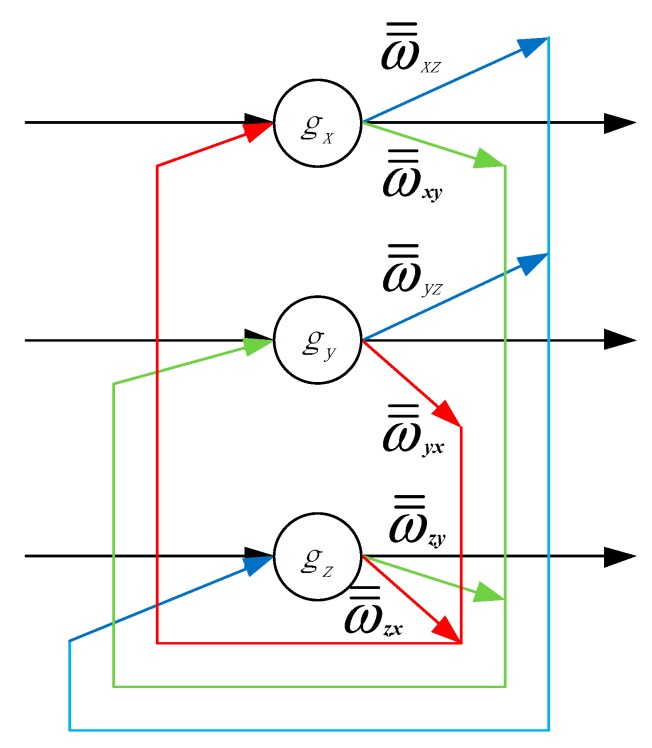
DHNN structure.

After Sorting and Classification, each grade of data is identified, and should be trained to decrease the omission and misinformation with DHNN. Moreover, the Discrete Hopfield Neural Network (DHNN) [34,35,36] is a feedback network, which has characteristics of single layer and output two-value. Its neuron outputs are 1 and −1, which express neurons in the activation and inhibition state, respectively. As seen in Figure 5, the DHNN structure is composed of three neurons.

This DHNN has no self-feedback network. While the DHNN layer is a neuron, which takes part in accumulating the product of the input information and the weight coefficient, and the output information is generated after application of the nonlinear function *h*, which is a simple threshold function. If the output information of the neuron is more than a threshold θ, the neuron is 1. Conversely, the neuron is −1. In addition, the input of this step is $S_{i,\;j}=\left\{s_{x},\;s_{y},\;s_{z}\right\}$, which is refreshed.

In view of a two-value neuron, its calculation Equation (26) is as follows:

(26) $$r_{j}=\overset{\;}{\underset{i}{\sum }}{\overset{=}{\text{ω}}}_{ij}s_{i}+k_{j},\;i=x,\;\text{y},\text{ z},\;j=x,\;\text{y},\text{ z},\;i\neq j$$

In Equation (26), *s_i_* is external input, and Equation (27) is written as follows:

(27) $$\{\begin{array}{c} g_{j}=1,\;\;\;r_{j}\geq \theta_{j}\\ g_{j}=-1,\;r_{j}<\theta_{j} \end{array}$$

One DHNN status is a set of output neuron information. For a DHNN with the output layer of *n^′^* neurons, there is the *n^′^* dimension vector at time *t^′^*. Equation (28) can be expressed as follows:

(28) $$G\left(t^{\prime }\right)={\left[g_{1}\left(t^{\prime }\right),\;g_{2}\left(t^{\prime }\right),\;\ldots ,\;g_{n^{\prime }}\left(t^{\prime }\right)\right]}^{T}$$

Since $g_{i}\left(t^{\prime }\right)\left(i=1,\;2,\;\ldots ,\;n\right)$ can be value 1 or −1, $G\left(t^{\prime }\right)$ with *n^′^* dimension vectors it has $2^{n^{\prime }}$ status. For normal DHNN nodes, the *j*th neuron can be expressed by $g_{i}\left(t^{\prime }\right)$. In other words, we assume that $g_{i}\left(t^{\prime }\right)$ is the status of the neuron *j* at time *t^′^*. Then, the status of the next time can be obtained from Equations (29) and (30) as follows:

(29) $$g\left(t^{\prime }+1\right)=h\left[r_{j}\left(t^{\prime }\right)\right]=\{\begin{array}{c} 1,\;r_{j}\left(t^{\prime }\right)\geq 0\\ -1,\;r_{j}\left(t^{\prime }\right)<0 \end{array}$$

(30) $$r_{j}\left(t^{\prime }\right)=\overset{n^{\prime }}{\underset{i=1}{\sum }}{\overset{=}{\text{ω}}}_{ij}s_{i}\left(t^{\prime }\right)+k_{j}-\theta_{j}$$

### 3.5. Output and Feedback

Now, most of the faulty sensors’ status is shown with more accuracy, but critical ones exist. Consequently, *S_i, j_* is divided into two groups. One with heavy faults is alarmed and reconditioned. The other is used in Equation (10) and comes through training, clustering and DHNN again until the upper limit is reached:

(31) $$\{\begin{array}{c} alarm_{2}\left(s_{i}\right)=S_{i,\;j},\;\;if\;j\geq c_{cl}-1\\ f^{5}\left(s_{i}\right)=S_{i,\;j}\;\;\;\;\;,\;\;if\;j<c_{cl}-1 \end{array}$$

### 3.6. Implementation of the Algorithm

*Step 1: Sample acquisition*. The original detected data including flue gas dimming extent, ambient temperature, and communication are obtained with each node.

*Step 2: Adjustment*. Two features are utilized. They are the root mean square of the filtered signal (FRMS) and the normalized summation of the positive amplitudes of the difference spectrum between the measured signal and the healthy one (NSDS). The original signals from the spectrum with separated time points are transformed into normal probabilities in a limited continuous time. Moreover, the term *Z_org_* has been introduced in Equation (3) of NSDS. When *Z_s_*(*t*) = 1, *Z_m_*(*t*) = 0. At this time, because the main communication equipment is bad, the sensor uses the standby equipment and is absolutely sure. Now it is identified to be the most serious problem, identified as *R*_4_.*Step 3: Fuzzy inference operator*. According to the fuzzy logic analysis, the original data are collected to calculate the fuzzy values about every node status, and determine the detected value set *O*_1_. At the same time, the heavy fault node data set is obtained. The data are obviously abnormal and selected as the first alarm and maintenance objects.

*Step 4: FCMA operator*. The detected set *O*_1_ is clustered to calculate the cluster centers. The fault diagnostic grades standard is obtained with the sorting and classification algorithm. Then, the nodes are selected, which belong to the lowest two grades *L_n_* and *L_n−1_* of all, to ensure the existence of faults.

*Step 5: DHNN optimization*. The clustering centers $\left\{e_{\gamma_{i}}\right\}$ are taken as a DHNN balance points, and the fault diagnosis index and index code is established. A network is created to have an evaluation, learn, simulation and classification on the detected value set *O*_1_.

*Step 6: Output and feedback*. According to the fault diagnostic grades standard, the detected value set *O*_1_ is divided into two groups. One with heavy faults is alarmed and reconditioned. The other one is returned to Step 3 and comes through training, clustering and DHNN again until the upper limit is reached.

**Figure 6:**
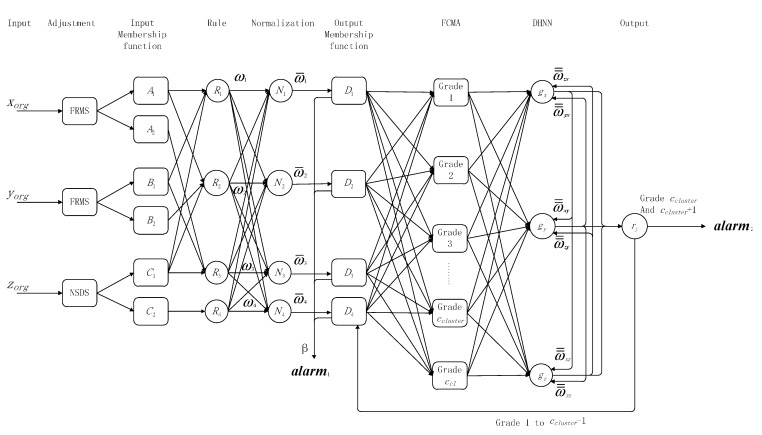
AF**-**DHNN structure.

*Step 7: Comparison*. AF-DHNN is compared with the data change rate and PSO algorithms. Finally, it suggests the advantages and disadvantages. The structure of AF-DHNN is shown in Figure 6.

## 4. Simulation and Analysis Results

Because establishing the AF-DHNN model needs less data at the time of input and determines balance points according to the actual situation, the simulation predication is improved, and the evaluation speed is faster.

### 4.1. Network Structure

The AF-DHNN system is a single fully connected network composed of three kinds of input data (Figure 6). Namely, the middle layer has three neurons: the flue gas dimming extent, environment temperature, main and standby communication modules. The output represents the recursive stable state of nodes with two values.

### 4.2. Sample Parameters

**Figure 7:**
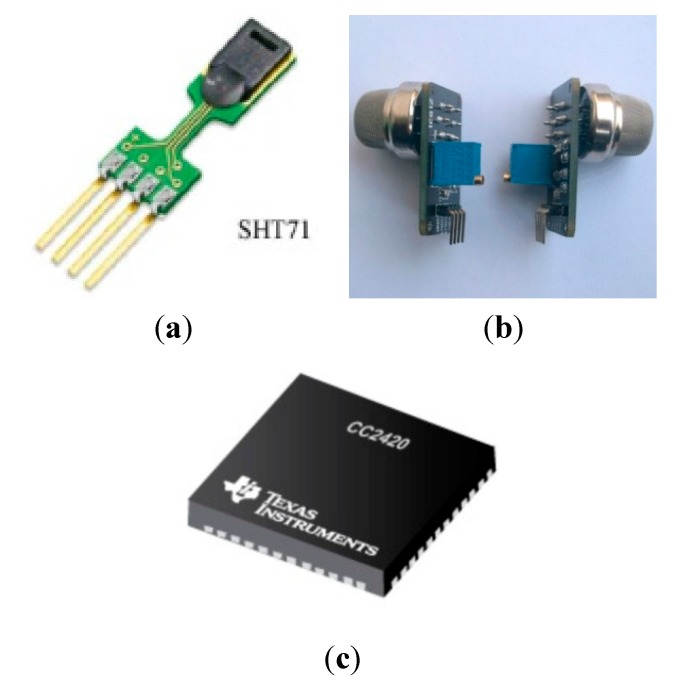
The modules chosen in the design. (**a**) temperature sensing module; (**b**) photoelectric smoke module; (**c**) radio frequency module.

**Figure 8:**
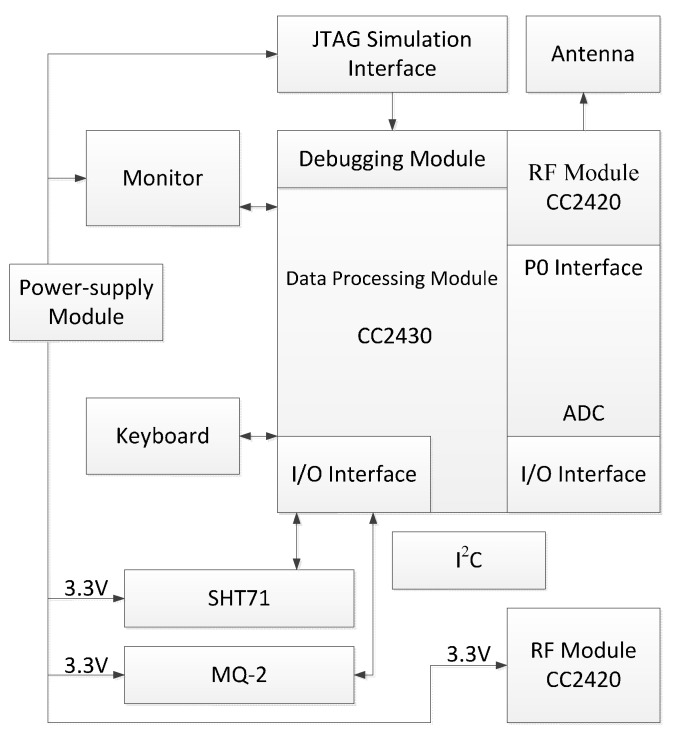
The whole design diagram of the system.

**Figure 9:**
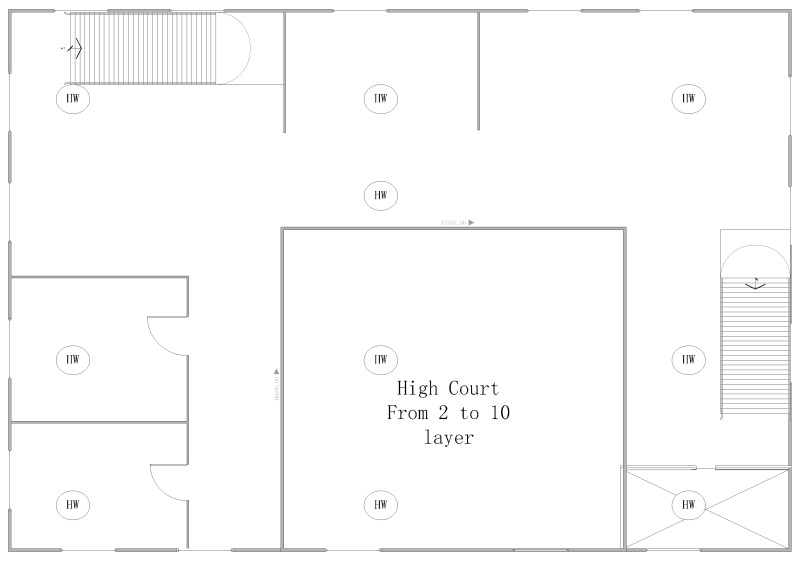
The Second to the 10th layer plane graph.

A 30 m tall high-rise building model was selected as the object of study. We assume that the building is located in a temperate city and has 10 internal layers, each with the same style, height and pattern. There are two stairwells, one elevator, and the atrium going through layers 1 to 10. There are windows to the outside around each layer in the architecture. The initial room temperature was 20 °C. The temperature universe is between 0 and 60 °C. The universe of the flue gas dimming extent parameter is between 0% and 100%. The wind from the air inlet at the main entrance can reach a speed of 10 m/s. In addition, 100 WSN fire detector nodes were set up in the building. All these nodes have the same configuration specifications, and the sensors are based on the ZigBee wireless communication protocol [37,38]. Furthermore, the SHT71 temperature detector, MQ-2 photoelectric smoke fire detector, and two CC2420 radio frequency modules, which are shown in Figure 7 and Figure 8, were adopted to perform the data acquisition. The building model is shown in Figure 9. The symbol 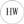 stands the vertical position of sensor nodes on each layer.

According to the characteristics of fire in buildings [39], the main factors related to the fire detection—flue gas dimming extent, ambient temperature, main and standby communication modules—were selected as input vectors. The output vectors were the classification of each node status (the first layer node serial number: 1 to 10; the second layer node serial number: 11 to 20; …; the 10th layer node serial number: 91 to 100).

Besides, the module data of each node showed small differences. Moreover, the number and location of the actual fault modules were generated randomly, and the sensitivity of the fault module was lower than the same product standards. Consequently, according to the membership function, assuming that a similar module’s data was lower than normal with 20% above, it was preliminarily identified as the faulty module. Also, if a node had more than 1 (including 1) module fault, the node was preliminarily determined as a faulty node. Next, the time state of each module of the experiment by normalization was utilized as fuzzy input. Then, the AF-DHNN algorithm model was compared with those of the data change rate and PSO algorithm, the items of which were of a typical fault node in the fault diagnosis accuracy, stability, and durability in combined testing time [40]. The parameter of the maximum number of iterations was set as 1000. In addition, to avoid a portion of the input not converging to equilibrium, but going into oscillation or a chaotic state near the zero value, the threshold 1.0e−4 was set for the detection of this condition.

### 4.3. Conclusion Analysis

#### 4.3.1. Fuzzy Input

**Figure 10:**
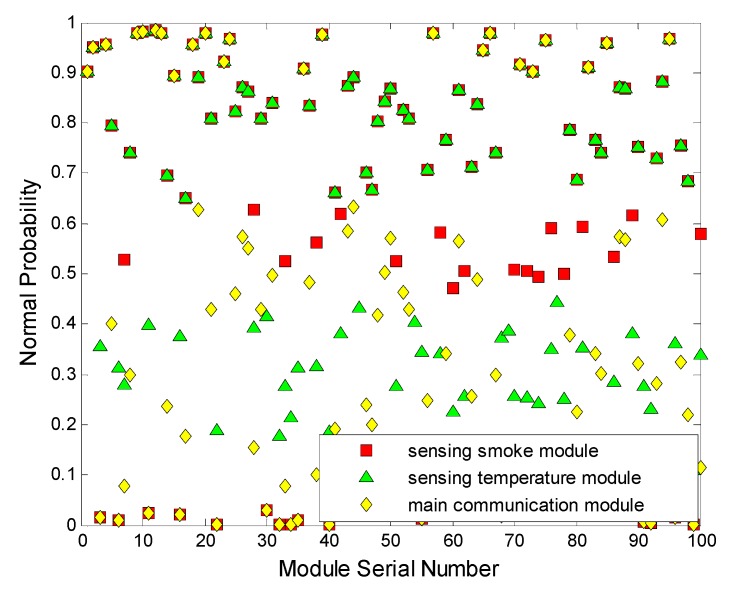
The original input.

**Figure 11:**
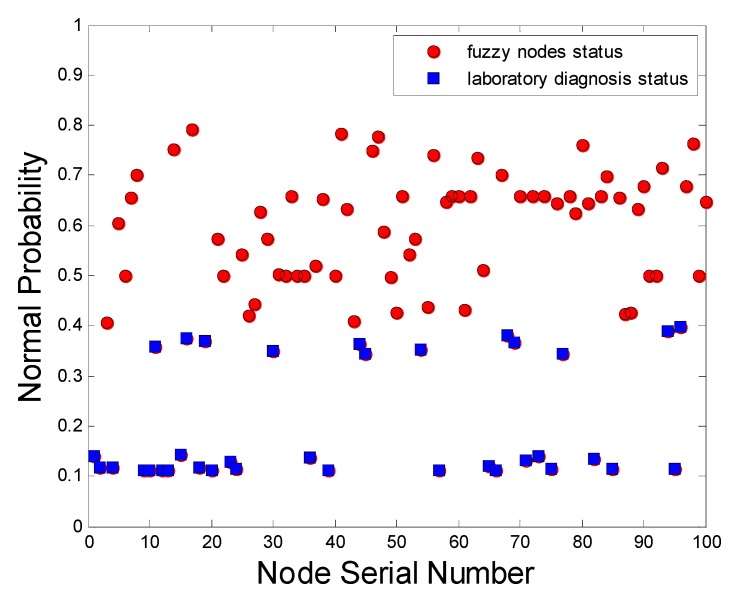
The actual state of all nodes and fuzzy inference state.

The experiment with T=10 s is actualized on each module in an independent combustion condition environment. As shown in Figure 10 and Figure 11, in the environmental range, the bottom is flammable and an air inlet, the top is exhaust outlet, and the test node is set in the median position.

As shown in Equation (32), we can obtain 11 obvious abnormal node positions based on the value of the node set in the range set O_*fuzzy−alarm*1_, which is obtained through the fuzzy inference operator and first time alarms:

(32) $${\text{O}}_{fuzzy‒alarm1}=[\begin{array}{c} 1110111110\\ 1111111111\\ 1101111111\\ 1111101111\\ 1110111111\\ 1111110111\\ 1111011111\\ 1100111111\\ 1111111111\\ 1111001111 \end{array}]$$

**Figure 12:**
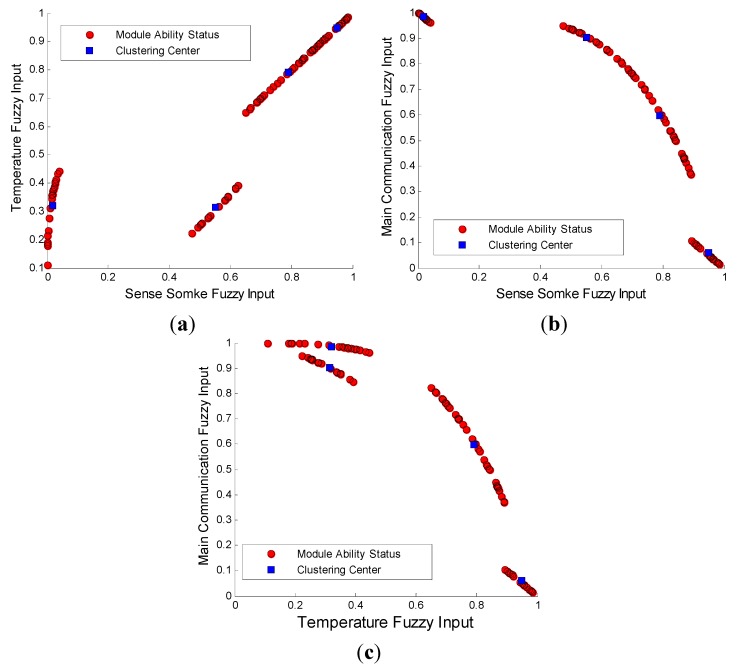
FCMA simulink.

At the same time, as shown in Figure 12, three kinds of modules of one node with FCMA are tested to find the clustering centers. Distinctly, four groups of notes are composed. $\epsilon \in \left[0,10^{-5}\right]$, θ∈(1,+∞), and *P* = 2.

According to what is shown above, the four clustering centers are:

$D_{1}\left(0.\;7898,\;0.\;7897,\;0.\;5973\right)$;
$D_{2}\left(0.\;5516,\;0.\;3143,\;0.\;9018\right)$;
$D_{3}\left(0.\;1680,\;0.\;3192,\;0.\;9845\right)$;
$D_{4}\left(0.\;9482,\;0.\;9482,\;0.\;0607\right)$.

Obviously, the center *D*_1_ is normal. In *D*_2_, many temperature sensing detector faults exist. A large number of smoke, temperature sensing detector faults are found in *D*_3_. The main fault in *D*_4_ is the main communication module problem.

#### 4.3.2. Diagnostic Method Performance Comparison

We sort adaptively according to Algorithm 1 to obtain the diagnosis grading system *L_n_*. As shown in Table 2, because the lowest level *L_n_* is zero, which lacks representation, two grades *L_n_* and *L_n−1_* are determined as the scope of faults.

**Table 2:** Diagnosis grading system.

Grade	Flue Gas Dimming Extent Grade	Flue Gas Dimming Extent	Temperature Grade	Temperature	Main Communication Grade	Main Communication
***L*_1_**	$L_{A_{1}}$	0.9482	$L_{B_{1}}$	0.9482	$L_{C_{1}}$	0.9845
***L*_2_**	$L_{A_{2}}$	0.7898	$L_{B_{2}}$	0.7897	$L_{C_{2}}$	0.9018
***L*_3_**	$L_{A_{3}}$	0.5516	$L_{B_{3}}$	0.3192	$L_{C_{3}}$	0.5973
***L*_4_**	$L_{A_{4}}$	0.1680	$L_{B_{4}}$	0.3143	$L_{C_{4}}$	0.0607
***L*_5_**	$L_{A_{5}}$	0	$L_{B_{5}}$	0	$L_{C_{5}}$	0

**Figure 13:**
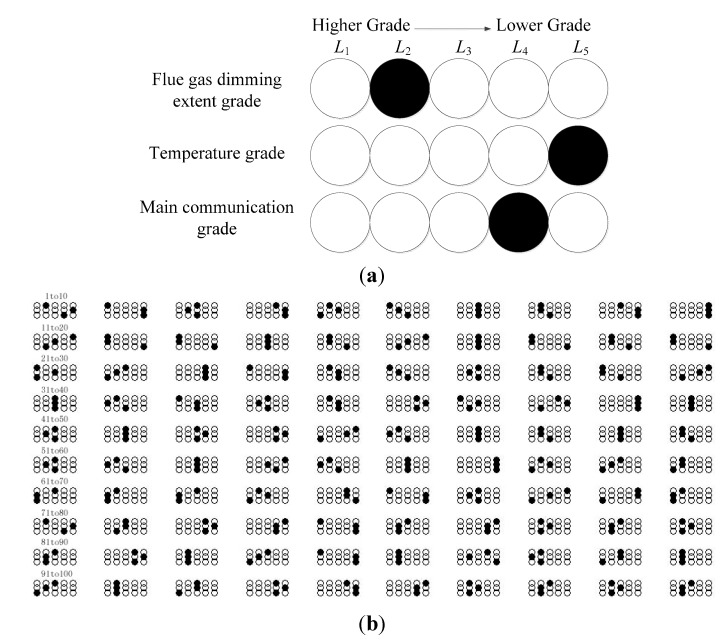
The output from AF-DHNN. (**a**) No.1 node’s status; (**b**) all nodes’ status.

As shown in Figure 13, DHNN is established and improved with the fuzzy status and diagnosis grading system of nodes and modules after 27 iterations. Besides, one of the 100 matrixes stands as a node’s status. In a matrix, the grade of flue gas dimming extent, which is utilized to determine faults, is the first line, temperature in the second, and the communication in the last. Also, the higher grades are on the left, the lower are on the right. Consequently, 11 faults of nodes from the conclusions of the fuzzy inference operator are found in the maintenance range by the AF-DHNN algorithm. It is convenient to test and repair the nodes with faults from the lowest grade to the higher.

As shown in Table 3, it is a list of the AF-DHNN output data distribution in each grade.

**Table 3:** The number of nodes.

Grade	Flue Gas Dimming Extent Grade	Flue Gas Dimming Extent	Temperature Grade	Temperature	Main Communication Grade	Main Communication
***L*_1_**	$L_{A_{1}}$	15	$L_{B_{1}}$	15	$L_{C_{1}}$	20
***L*_2_**	$L_{A_{2}}$	29	$L_{B_{2}}$	29	$L_{C_{2}}$	20
***L*_3_**	$L_{A_{3}}$	34	$L_{B_{3}}$	31	$L_{C_{3}}$	31
***L*_4_**	$L_{A_{4}}$	10	$L_{B_{4}}$	6	$L_{C_{4}}$	13
***L*_5_**	$L_{A_{5}}$	12	$L_{B_{5}}$	19	$L_{C_{5}}$	16

In the existing methods of fault diagnosis on WSN sensors [41,42,43,44], two common ones are introduced and compared with AF-DHNN algorithm. One is data change rate test method.

A continuous 10 s length test is set as a sample. As shown in Equation (33), we compute the data change rate *Rate*(*k*):

(33) Rate(k)=(Data(k)−Data(k−1))/T

where *T* is the sampling period. *Thr*(*A*) *and Thr*(*B*) are the boundaries of the pick data zone, which establish the suitable position and width of the pick data zone. *SectN* is the number of detection times.

The rate of data change is sorted according to its size. As shown in Equation (34), we remove the maximum and minimum value and take the rest to be the average of the initial data:

(34) $$RateAve\left(k\right)=\overset{SectN-2}{\underset{k=1}{\sum }}Rate\left(k\right)/\left(SectN-2\right)$$

As shown in Equation (35), if k>SectN, recursive average data change rate every time:

(35) RateAve(k)=RateAve(k−1)+(Rate(k)−Rate(k−SectN−1))/SectN

As shown in Equations (36) and (37), the pick data zone *Thr*(*A*) and *Thr*(*B*) are computed:

(36) Thr(B)=max(|RateAve(k−1)|, …, |RateAve(k−9)|)

(37) Thr(A)=3*Thr(B)

**Figure 14:**
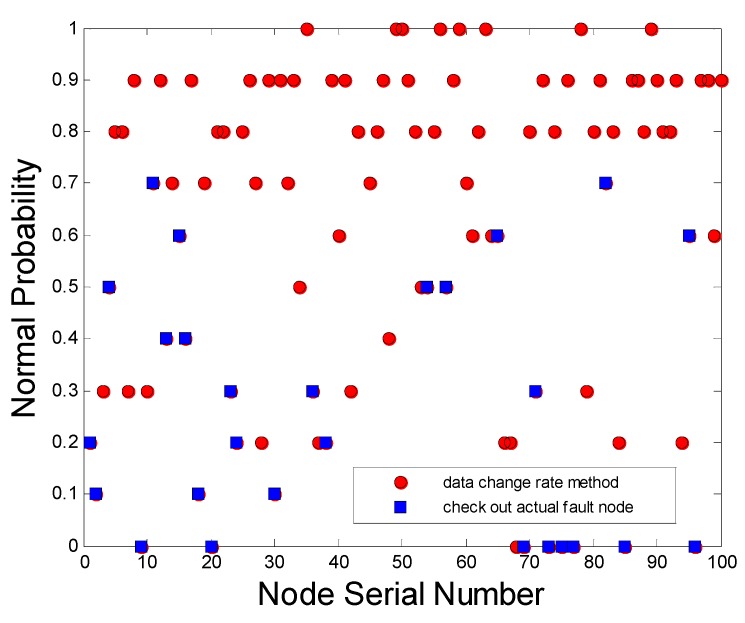
The accuracy rate of diagnosis of the rate of change of data test.

As shown in Figure 14, if more than 20% (more than two groups) are outside the scope of the pick data zone *Th*(*A*) and *Thr*(*B*) in every 10 groups of sample data, the node is determined to be faulty. As a result, the blue squares are accurate detection; the red circles are the other nodes’ status. Obviously, some of the nodes with low normal probability are lost detected. Because of the selected pick data zones are rigid, parts of the faults are not included.

Another common method is the PSO test. The first 100 nodes’ data are collected to produce 10 s length samples. The fitness of every particle is computed. The available maximum flue gas dimming extent, temperature and main communication status compose the initial *gbest*. The other particles determine their individual extreme *pest* to be their initial positions. After iteration, the global extreme *gbest^k^* are obtained and every particle selects an extreme *pbest^k^* according to its own flight records. Consequently, the refresh speed and position of every particle, and the global extreme *P_g_* is obtained in the range of iteration. According to the preset status threshold range: the flue gas dimming extent range is (5%, 100%), temperature range is (25 ℃, 60 ℃), and the communication is (0, 1). In Equations (38) and (39), the particle position and velocity updated equations are shown.

(38) $$g_{id}^{k+1}=\text{ϖ}g_{id}^{k}+c_{1}r_{1}\left(p_{id}-z_{id}^{k}\right)+c_{2}r_{2}\left(p_{gd}-z_{id}^{k}\right)$$

(39) $$z_{id}^{k+1}=z_{id}^{k}+g_{id}^{k+1}$$

where the particles are expressed as $Z_{i}=\left(z_{i1,}z_{i2,}\ldots ,z_{iD}\right)$; the best positions are $P_{i}=\left(p_{i1,}p_{i2,}\ldots ,p_{iD}\right)$; the velocities are $V_{i}=\left(g_{i1,}g_{i2,}\ldots ,g_{iD}\right)$. Also, the i=1,2,…,m;d=1,2,…,D; k is iterations; *r*_1_ and *r*_2_ are random numbers in the range [0,1]. The maximum iteration number *k_max_* is 100, the inertia weight is random in the range [0.9, 1.2], and acceleration factors *c*_1_ and *c*_2_ are both 2.

**Figure 15:**
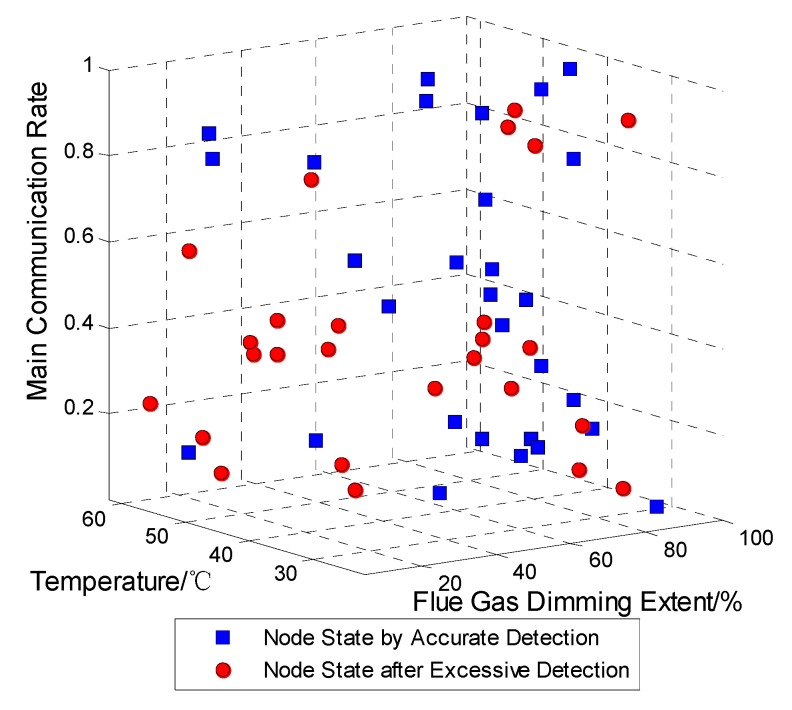
The accuracy rate of diagnosis of PSO test method.

As shown in Figure 15 as a result, two kinds of nodes are checked out by PSO method. One is accurate detection, which expresses some of the nodes with actual faults; the others called excessive detection are not failures but normal sensors. Visibly, the nodes with doubtful faults after detection are many more than the actual faults, Also, the distributed computation does not incur excessive communication cost and energy consumption.

As shown in Table 4, the diagnosis accuracy rate of test method of AF-DHNN is far higher than that of the other two methods. Moreover, the number of diagnosis times and diagnosis of invalid nodes are less. Hence, the method has obvious advantages in practical applications [45]. Data change rate test method mainly depends on the testing data of each detector and the data analysis was less. Its pick data zone is set based on the average detection data, which is highly dependent on the stability of the detection conditions. Therefore, it does not apply to a fire environment with large changes.

**Table 4:** Sensor faults diagnosis accuracy.

Project	The Actual Number of Failure	AF-DHNN			Data Change Rate			PSO		
		The Accuracy Rate of Diagnosis	Maintenance Number (Piece)	The Time of Diagnosis (s)	The Accuracy Rate of Diagnosis	Maintenance Number (Piece)	The Time of Diagnosis (s)	The Accuracy Rate of Diagnosis	Maintenance Number (Piece)	The Time of Diagnosis (s)
Photoelectric smoke module	20	100%	22	10	80%	28	100	85%	32	10
Temperature sensing module	23	95.65%	25	10	73.91%	44	100	86.95%	47	10
The main communication module	26	100%	29	10	76.92%	32	100	76.92%	35	10
Node	35	97.14%	40	13.3	77.14%	53	124.2	82.85%	55	15.7

The PSO algorithm is easy to implement, it has fast convergence speed, and the accurate diagnosis rate is higher. However, it is not suitable for environments with a large number of WSN nodes and high fault rate conditions. Besides, there are limitations in high-rise buildings and large span space environments.

**Figure 16:**
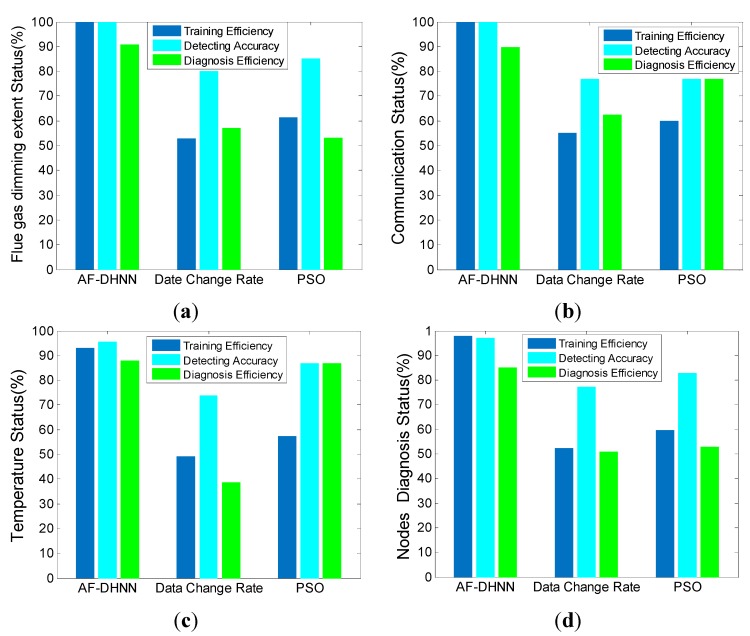
The diagnosis performance comparison in normal environment. (**a**) flue gas dimming extent status; (**b**) communication status; (**c**) temperature status; (**d**) nodes diagnosis status.

As shown in Figure 16, two new parameters are introduced. One is training efficiency [46,47]. It takes the number of training processing sessions of the AF-DHNN as 100%, which achieves the top efficiency, but those of the other methods are lower because they have more training requirements. The distinction is that the AF-DHNN has an advantage in the form of weights, which combine the membership functions, rules, and normalization exactly. Besides, the network structure is more quickly transformed with the characteristics of input data than with the other two methods. Moreover, the characteristics are obtained with FRMS and NSDS, which completely turn the continuous values into discrete ones, but without distortion. It also accelerates the training.

The other one is diagnosis efficiency [48,49]. It expresses the regulation between detected accuracy and the ones selected by a method. Most times, a normal processing is diagnosed from more sensors than the exact one. The miscarriages are less, the energy consumption is lower, and the judgment capability is stronger. In this paper, the proposed method has advantages such as effective detection, which can select the fault sensors accurately, but let the others off. Because the FCMA and Sorting and Classification algorithm establish a diagnosis grading system, the detected data does not have to be compared with the other sensors one by one. Then, the data are divided and come through the DHNN, where it is transformed with iterations. Besides, the grades and weights of DHNN are not fixed, until the detected result is stable or the iterations reach the superior limit. Also, the twice-alarmed mechanism plays a part in the processing, which accelerates the iterations.

#### 4.3.3. Performance Comparison in a Fire

The real working status can be obtained after AF-DHNN diagnosis analysis. In the performance comparison, the intact status and the former three diagnosis methods with real sensors status are taken into a fire environment for verification of the specific performance. Because of the effects of the smoke plume, there exists a maximum flue gas dimming extent and temperature in the second floor in the building, which is the most representative.

**Figure 17:**
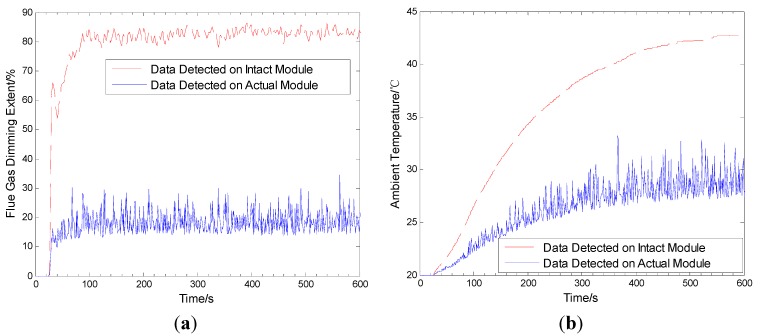
The real and detected average environmental parameters in fire. (**a**) flue gas dimming extent change; (**b**) ambient temperature change.

It is easy to understand that not each node expresses a wide gap, because some nodes have few faults. They have been included in the higher grades to the faults. In contrast, according to Figure 13, the 16th node with visible faults on the second floor can be selected to reveal how much the performance gap between the above three kinds of method is. Consequently, the true environmental parameters and the detected data, which are the combination of 1000 experiments, are shown in Figure 17. Then, it can be seen that the two modules with different functions have faults and particular characteristics, in which the gap between true and faulty nodes becomes larger and larger. They are both separated into initial, fast developing, and violent stages. One of them is the flue gas dimming extent change, at which the violent stage lasts longer than the other two. The other is the ambient temperature change, at which the development stage is the longest of all. Indeed, the next analyses about the detection at each stage can be expressed clearly. In addition, the communicaion status here is like in a normal enviroment, so it need not to be introduced again.

**Figure 18:**
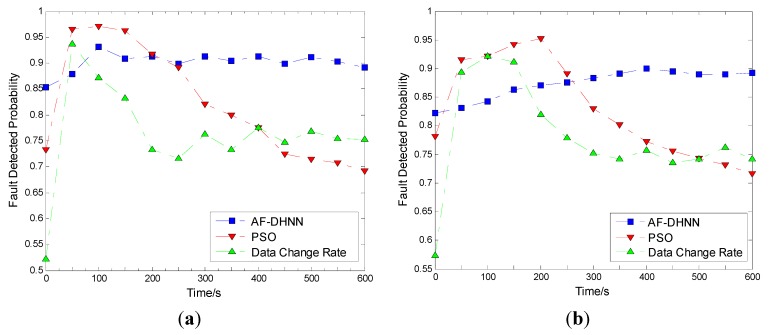
Performances of diagnosis methods compared in fire. (**a**) flue gas dimming extent change; (**b**) ambient temperature change.

The performances of the diagnosis methods are compared on the 16th node in Figure 18. The changes of fault detected probabilities with time are also separated into the former three stages.

Then, the analysis is as follows: firstly, it is about accuracy [50,51]. The fault detection probabilities with time are utilized to reveal the accuracy ability of the related module. After computation, the average fault detection probability of AF-DHNN is 0.9014; that of the data change rate method is 0.7620; and for the PSO method it is 0.8214. Furthermore, the AF-DHNN method is the most effective at the initial stage and violent stage, because the FCMA establishes a self-adaptive sorting system with time, and the grades are regulated with environment changes, regardless of whether there is a fire or not. Likewise, the AF-DHNN method is effective at the development stage, but the PSO and data change rate methods are a little more effective. One reason is the data change rate relies on the pick data zones, which are determined with the history data. While the new data are changed so fast that they are out of the zones, the system considers that faults appear. Another reason is the PSO method, which is utilized to detect the fire in an unbalanced environment, for the heated and smoked portion of the space, and the samples obtained with the global extreme. Besides, the PSO acquires samples in a shorter time than the other two, so it is more sensitive in a fast changing environment, but the development stage is exceedingly short in a whole fire. In this item, the AF-DHNN is more effective.

Secondly, it is about stability [52,53]. It is obvious that the AF-DHNN method is smoother than the other two. After computation, the fault variance detected probability of AF-DHNN is 0.0004; of the data change rate method it is 0.0091; and for the PSO method it is 0.0115. Furthermore, the AF-DHNN is steady, because the fuzzy controller is so inclusive that the membership functions can be set up to deal with a massive situation in advance, but with little effect on detection. In contrast, the data change rate and the PSO method focus on the transformations, but are insufficient to work in a smooth environment like the initial and violent stages effectively. Besides, the former focuses on time change, and the later focuses on the globe change at single time points. Hence, the AF-DHNN is also more effective.

Thirdly, it is about durability [54,55]. In this item, the modality of energy consumption is essential. The AF-DHNN is a mixed model. The distributional nodes are just responsible for fuzzy inference collection with FRMS and NSDS. Besides, the FCMA, sorting and classification, and DHNN algorithms are carried out by centralization. Likewise, the data change rate arranges a few computations on each node. In contrast, all local extremes are selected to be compared on nodes after every time detect point by the PSO method, so the energy consumption huge with the PSO method. In a word, it is shown that the curve standing PSO method moves down at the violent stage acutely, but the other two do not. Therefore, the AF-DHNN and data change rate methods are better than the rest in this item. In summary, it is obvious that the AF-DHNN with fuzzy and neural network algorithms has more advantages than the data rate of change and PSO algorithms.

## 5. Conclusions

The AF-DHNN method [56] does not depend on the categories of the sensors and is a method suitable for high-rise buildings and environments with large spans, large spaces and multi-rooms. The AF-DHNN model needs less data at the input time for establishing it for the fuzzy input data, which can accurately define the comprehensive situation of the environment in buildings. The spare time is arranged for synchronization. In recent years, with the enhanced distributed computation and node power load ability, we can modify the membership functions and fuzzy rules, and establish Hopfield networks with more neurons at the middle layer which can cause a large increase in the same node representing different fault states [57,58]. However, with the improvement of the thermal imaging and remote sensing technology [59,60], further research is necessary to prove whether the AF-DHNN method is applicable to very sensitive environmental changes or not.
